# Engagement of Family in a Goal Setting Strategy: Impact Upon 30-Day Hospital Readmissions

**DOI:** 10.1093/geroni/igab046.1447

**Published:** 2021-12-17

**Authors:** Marie Boltz, Ashley Kuzmik, Barbara Resnick, Irene Best, Jacqueline Mogle

**Affiliations:** 1 Pennsylvania State University, University Park, Pennsylvania, United States; 2 University of Maryland School of Nursing, Baltimore, Maryland, United States; 3 Penn State University, University Park, Pennsylvania, United States

## Abstract

Family-centered Function-focused Care (Fam-FFC) works with family caregivers as care partners in the assessment, function-promoting goal setting, implementation, and evaluation of goal attainment during hospitalization and immediate post-acute period. ANCOVA technique examined the preliminary impact of Fam-FFC upon 30-day hospital readmissions and logistic regression tested the association of goal attainment, measured with the Goal Attainment Scale (GAS) with 30-day hospital readmissions. The majority of the patients were Black (50%), female (62%), had a mean age of 81.6 (SD=8.4), mean Barthel Index of 60.29 (SD=27.7), and mean MoCA of 10.67 (SD=7.0). Goals represented six main categories: mobility, cognition, self-care, toileting, sleep, and pain management. Patients in the intervention group had less 30-day hospitalizations (F= 4.6, p=.033) and goal attainment was significantly associated with less recidivism (B=.179, Wald= 2.8 (1), p= .045). FamFFC shows promise in reducing 30-day hospital readmissions; results support the contribution of family engagement and use of GAS

